# Supplementary material for: Divacancy and resonance level enables high thermoelectric performance in n-type SnSe polycrystals
Source: Nat Commun. 2024 May 18;15:4231. doi: 10.1038/s41467-024-48635-0 (PMC11102544; doi:10.1038/s41467-024-48635-0)
Supplement: Supplementary file 1 — Supplementary Information [file 41467_2024_48635_MOESM1_ESM.pdf]

# Supporting Information

# Divacancy and Resonance Level Enables High Thermoelectric Performance in *n*-Type SnSe Polycrystals

**Yaru Gong,[1, #] Wei Dou,[1, #] Bochen Lu,[2, #] Xuemei Zhang,[3] He Zhu,[1] Pan Ying,[1] Qingtang Zhang,[1] Yuqi Liu,[1] Yanan Li,[1] Xinqi Huang,[1] Muhammad Faisal Iqbal,[1] Shihua Zhang,[1] Di Li,[4] Yongsheng Zhang,[5 *] Haijun Wu,[2 *] Guodong Tang[1, *]**

[1] National Key Laboratory of Advanced Casting Technologies, MIIT Key Laboratory of Advanced Metallic and Intermetallic Materials Technology, Engineering Research Center of Materials Behavior and Design, Ministry of Education, Nanjing University of Science and Technology, Nanjing, 210094, China

[2] State Key Laboratory for Mechanical Behavior of Materials, Xi'an Jiaotong University, Xi'an, 710049, China

[3] School of Physics and Electronic Information Engineering, Engineering Research Center of Nanostructure and Functional Materials, Ningxia Normal University, Guyuan, Ningxia 756000, China

[4] Key Laboratory of Materials Physics, Institute of Solid State Physics, Chinese Academy of Sciences, Hefei 230031, China

[5] Advanced Research Institute of Multidisciplinary Sciences, Qufu Normal University, Qufu, Shandong Province, 273165, China

[#] These authors contributed equally to this work

[*] To whom correspondence should be addressed

Email: tangguodong@njust.edu.cn (G. D. Tang)

Email: wuhaijunnavy@xjtu.edu.cn (H. J Wu)

Email: yshzhang@theory.issp.ac.cn (Y. S. Zhang)

## Lorenz number calculation in details:

In general, the total ($\kappa$) consists of the electronic thermal conductivity ($\kappa_e$) and lattice thermal conductivity ($\kappa_L$). The electronic part $\kappa_e$ is directly proportional to the electrical conductivity $\sigma$ through the Wiedemann-Franz relation, $\kappa_e = L\sigma T$, where $L$ is Lorentz number and its value is calculated by SPB model. The Lorenz number can be given as: [1, 2]

$$L = \frac{k_B^2}{e^2}\left(\frac{(r+3)F_{r+2}(\eta)}{(r+1)F_r(\eta)} - \left[\frac{(r+2)F_{r+1}(\eta)}{(r+1)F_r(\eta)}\right]^2\right) \quad (1)$$

For the Lorenz number calculation, we should get reduced Fermi energy $\eta$ firstly. The calculation of $\eta$ can be derived from the measured Seebeck coefficients by using the following relationship:

$$S = \pm\frac{k_B}{e}\left(\frac{(r+2)F_{r+1}(\eta)}{(r+1)F_r(\eta)} - \eta\right) \quad (2)$$

where $F_n(\eta)$ is the $n$–th order Fermi integral,

$$F_n(\eta) = \int_0^\infty \frac{\chi^n}{1+e^{\chi-\eta}}d\chi \quad (3)$$

where $e$ is the electron charge, $k_B$ is the Boltzmann constant, $h$ is the Planck constant, $r$ is the scattering factor. Here, $r$ is 0 since acoustic phonon scattering has been assumed as the main carrier scattering mechanism near room temperature (RT). Lorentz number can be obtained by combining equations (1), (2) and (3).

**Supplementary Table 1.** Rietveld refinement details of $SnSe_{0.92}$+0.03$WCl_6$.

| Atom | Site | x | y | z | Occupancy | FWHM(111) |
|---|---|---|---|---|---|---|
| Sn | 4c | 0.1187 | 0.2500 | 0.0988 | 0.8451 | 0.2368 |
| W | 4c | 0.1187 | 0.2500 | 0.1124 | 0.0157 | |
| Se | 4c | 0.8520 | 0.2500 | 0.4867 | 0.7665 | |
| Cl | 4c | 0.8710 | 0.2500 | 0.4517 | 0.1246 | |

**Supplementary Table 2.** Lattice parameters for $SnSe_{0.92}$ + x $WCl_6$ (x = 0, 0.01, 0.02, 0.03, 0.04).

| Compositions | a（Å） | b（Å） | c（Å） | Volume（$Å^3$） |
|---|---|---|---|---|
| x = 0 | 11.48539 | 4.15977 | 4.45076 | 212.64 |
| x = 0.01 | 11.49886 | 4.16014 | 4.44020 | 212.41 |
| x = 0.02 | 11.44983 | 4.13438 | 4.41513 | 209.00 |
| x = 0.03 | 11.43914 | 4.13616 | 4.41007 | 208.66 |
| x = 0.04 | 11.44082 | 4.11962 | 4.42932 | 208.76 |

**Supplementary Table 3.** Room temperature carrier concentration of $SnSe_{0.92}$ + x $WCl_6$ (x = 0.01, 0.02, 0.03, 0.04) along the pressing direction.

| | Carrier Concentration, *n* ($10^{18}$ $cm^{-3}$) |
|---|---|
| $SnSe_{0.92}$ | 0.125 |
| x=0.01 | 4.174 |
| x=0.02 | 0.3513 |
| x=0.03 | 0.1909 |
| x=0.04 | 0.1529 |

**Supplementary Table 4.** The calculated and measured densities for $SnSe_{0.92}$ + x $WCl_6$ (x = 0, 0.01, 0.02, 0.03, 0.04).

| Compositions | Actual Density ($\rho$, g/cm$^3$) |
|---|---|
| x=0 | 6.04 |
| x=0.01 | 6.02 |
| x=0.02 | 5.99 |
| x=0.03 | 5.97 |
| x=0.03 | 5.98 |

**Supplementary Figure 1.** Comparison of the G($r$) patterns in the real space (2–30 Å).

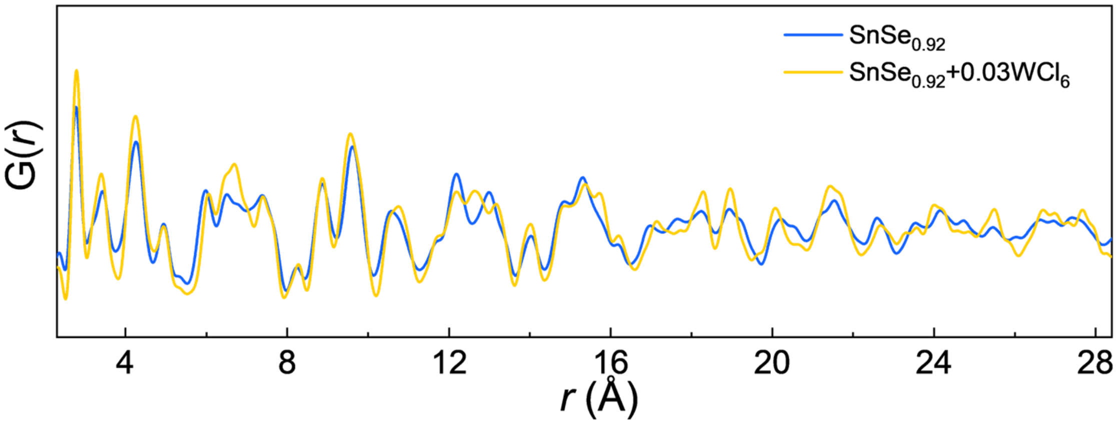

**Supplementary Figure 2.** Temperature dependence of (**a**) carrier concentration (n), (**b**) carrier mobility (μ) for $SnSe_{0.92}$+0.03$WCl_6$ and $SnSe_{0.92}$+0.04$WCl_6$.

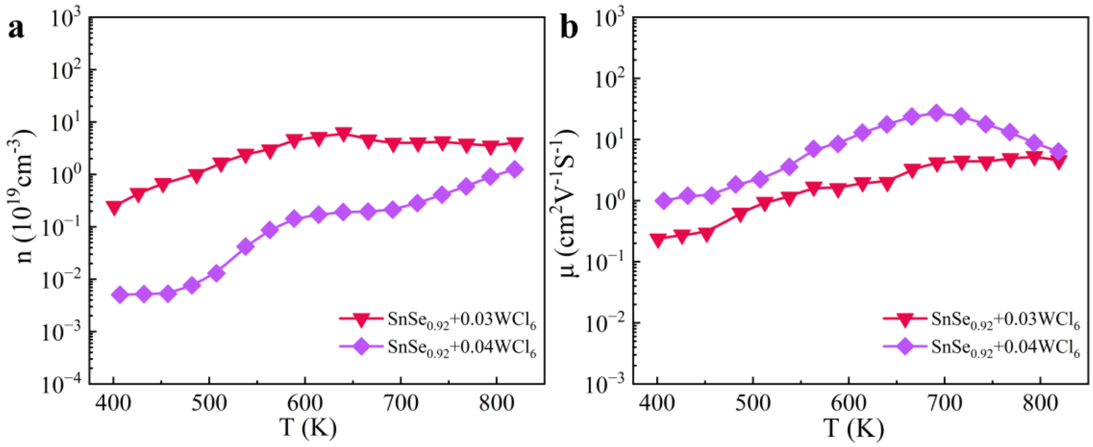

**Supplementary Figure 3.** Comparison of Seebeck coefficient of $SnSe_{0.92}$+0.03$WCl_6$ with other reported n-type SnSe-based materials.

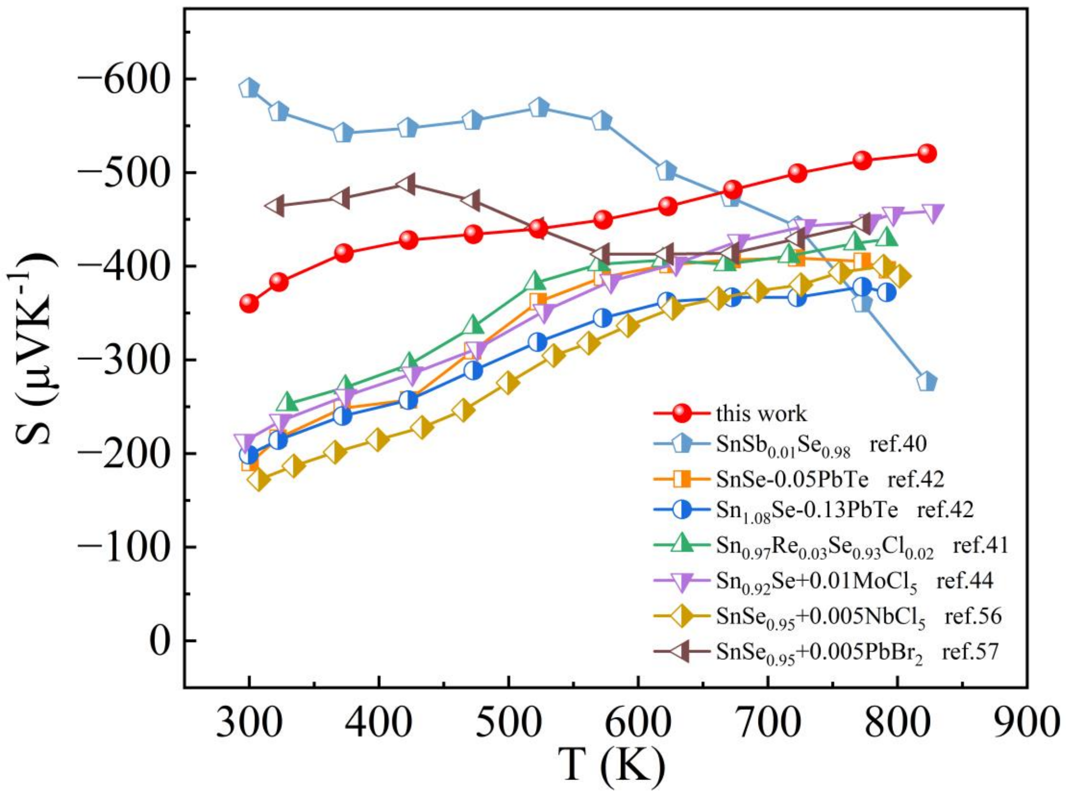

**Supplementary Figure 4.** Calculated density of states of $Sn_{0.946}W_{0.054}Se_{0.92}$ (the blue line) and pristine SnSe (the black line). The dashed line represents the Fermi energy level.

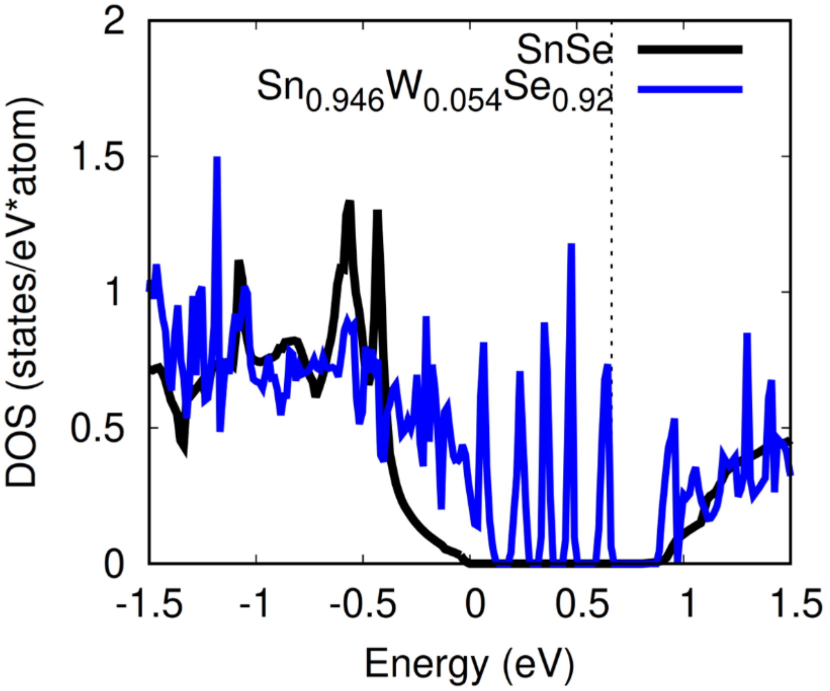


To elucidate the decline in Seebeck coefficient with increasing temperature in the elevated temperature range for the $SnSe_{0.92}$+$0.04WCl_6$ compound, we carried out the density of states (DOS) calculations of a high W concentration in SnSe (~5% W doping, denoted as $Sn_{0.946}W_{0.054}Se_{0.92}$ in Fig. S4). Our analysis revealed that at a lower W concentration (such as 3%), a limited number of discrete defect energy levels manifest within the band gap. These discrete defect energy levels within the gap do not contribute to the electron transport, and the band gap remains akin to that of the pristine SnSe. However, at higher W concentrations (>4%), certain discrete defect energy levels within the band gap coalesce into the energy band (from 0 eV to ~0.5 eV in Fig. S4). This energy band obviously involves in the carrier transport. Consequently, the valence band maximum (VBM) shifts to the position ~0.5 eV and the band gap contracts to ~0.3 eV. As a result, the substantial reduction in the band gap due to high W doping instigates a bipolar effect at elevated temperatures, leading to a decline in the Seebeck coefficient.

**Supplementary Figure 5.** Room temperature $S$ as a function of carrier concentration.

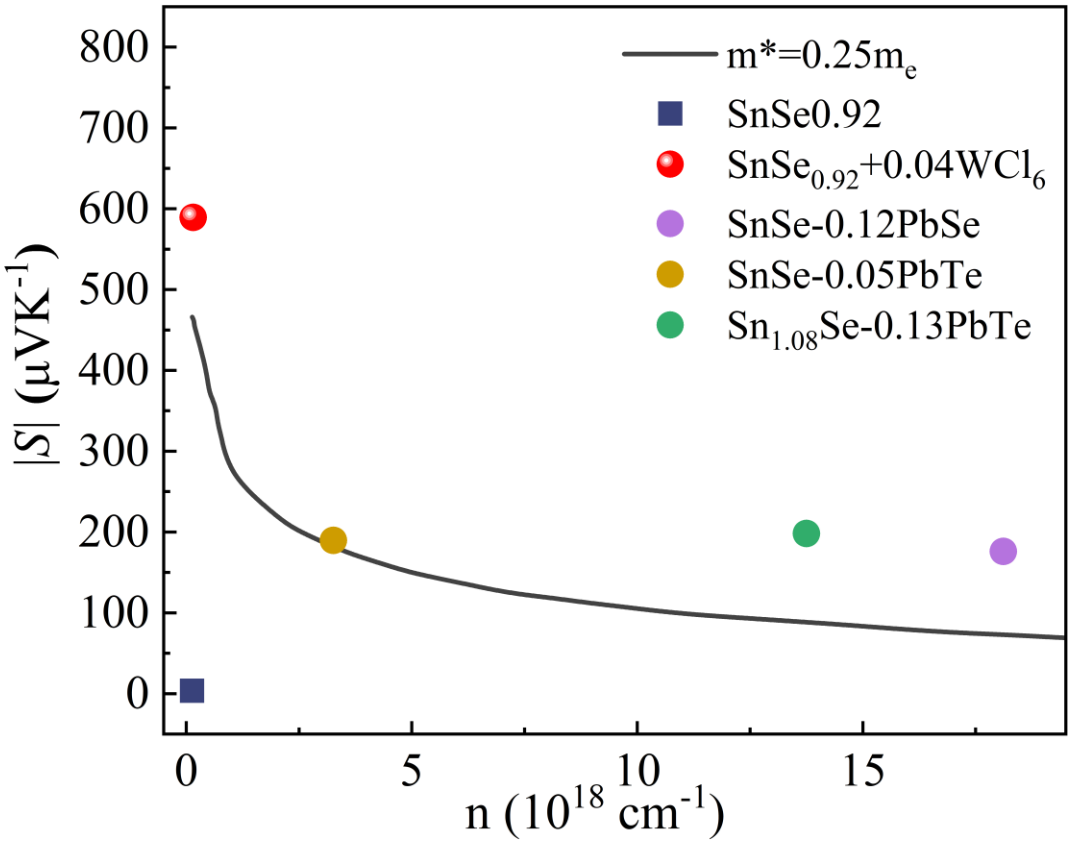

**Supplementary Figure 6.** Crystal structures of SnSe. (**a**) primitive cell, (**b**) perfect supercell, (**c**) $SnSe_{0.92}$ with Se vacancy.

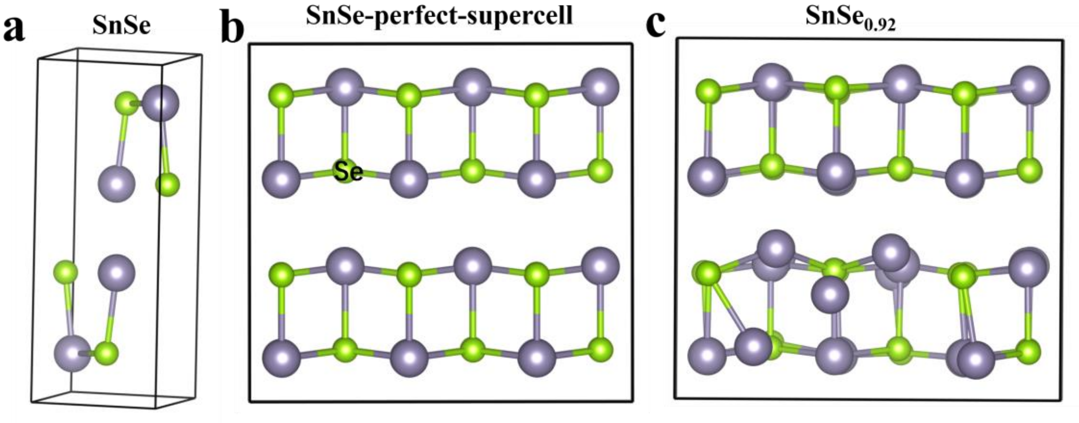

**Supplementary Figure 7.** Projected density of states of (**a**) SnSe, (**b**) $SnSe_{0.92}$ and (**c**) $Sn_{0.963}W_{0.027}Se_{0.92}$. The dashed line represents the Fermi energy level.

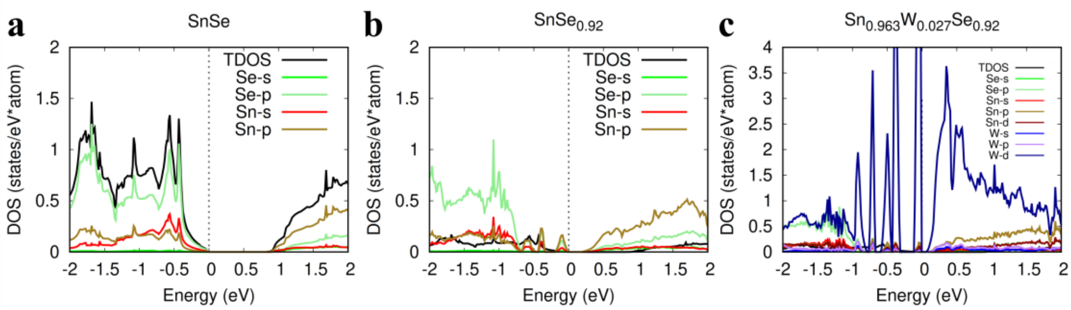

**Supplementary Figure 8.** Comparison of power factor of $SnSe_{0.92}$+$0.03WCl_6$ with other reported n-type SnSe-based materials.

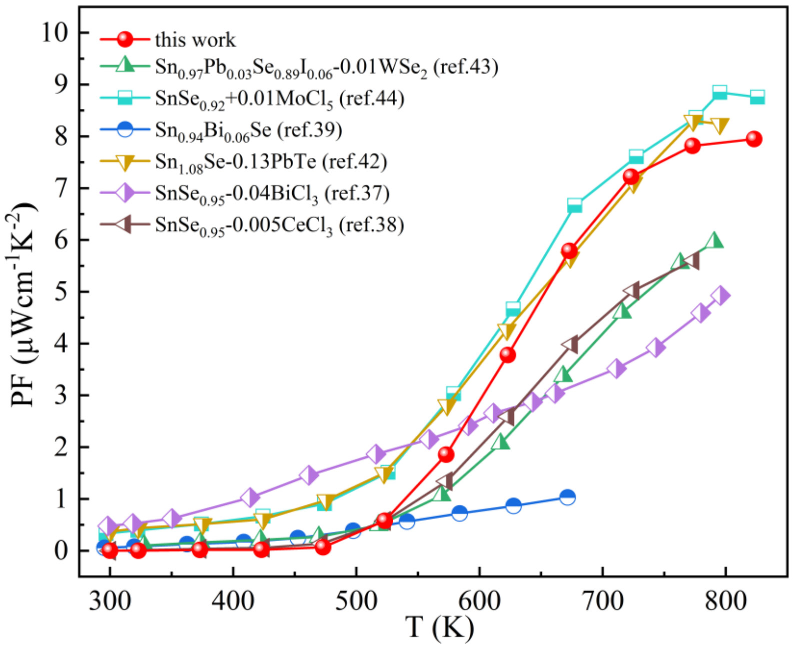

**Supplementary Figure 9.** Thermal transport properties as a function of temperature for $SnSe_{0.92}$ + x $WCl_6$ . (**a**) Electronic thermal conductivity ($\kappa_e$), (**b**) Lorenz number.

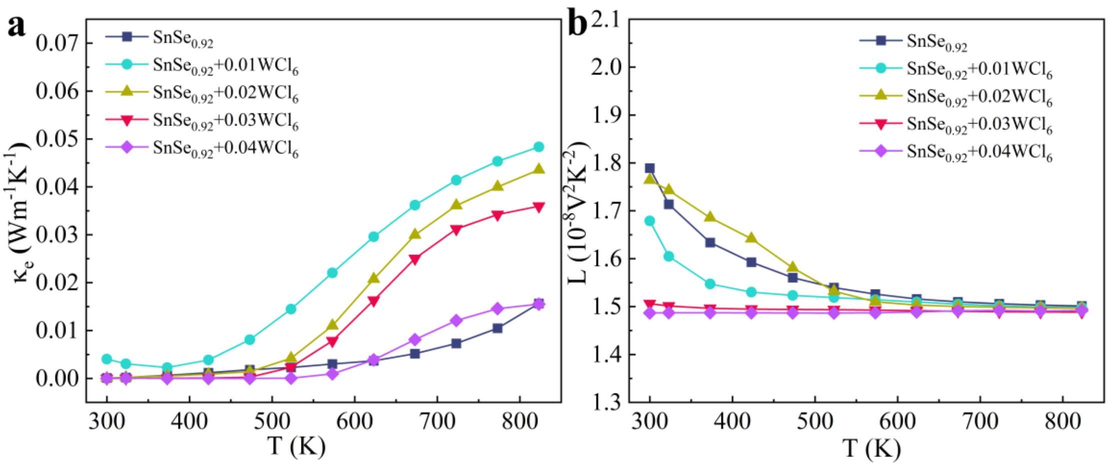

**Supplementary Figure 10.** Microstructures and grain sizes comparison for (**a**) $SnSe_{0.92}$ + $0.03WCl_6$ and (**b**) $SnSe_{0.92}$ + $0.04WCl_6$ samples.

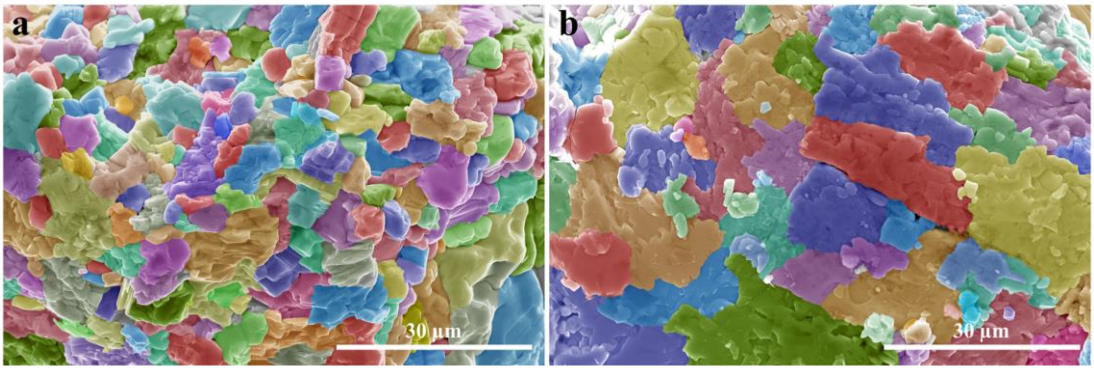

**Supplementary Figure 11.** HAADF images and EDS analysis nanoprecipitates of $SnSe_{0.92}$ + $0.03WCl_6$. (**a, b**) HAADF images of the matrix and nanoprecipitates. (**c, d**) Quantitative EDS analysis of the red area of (**a**) and (**b**).

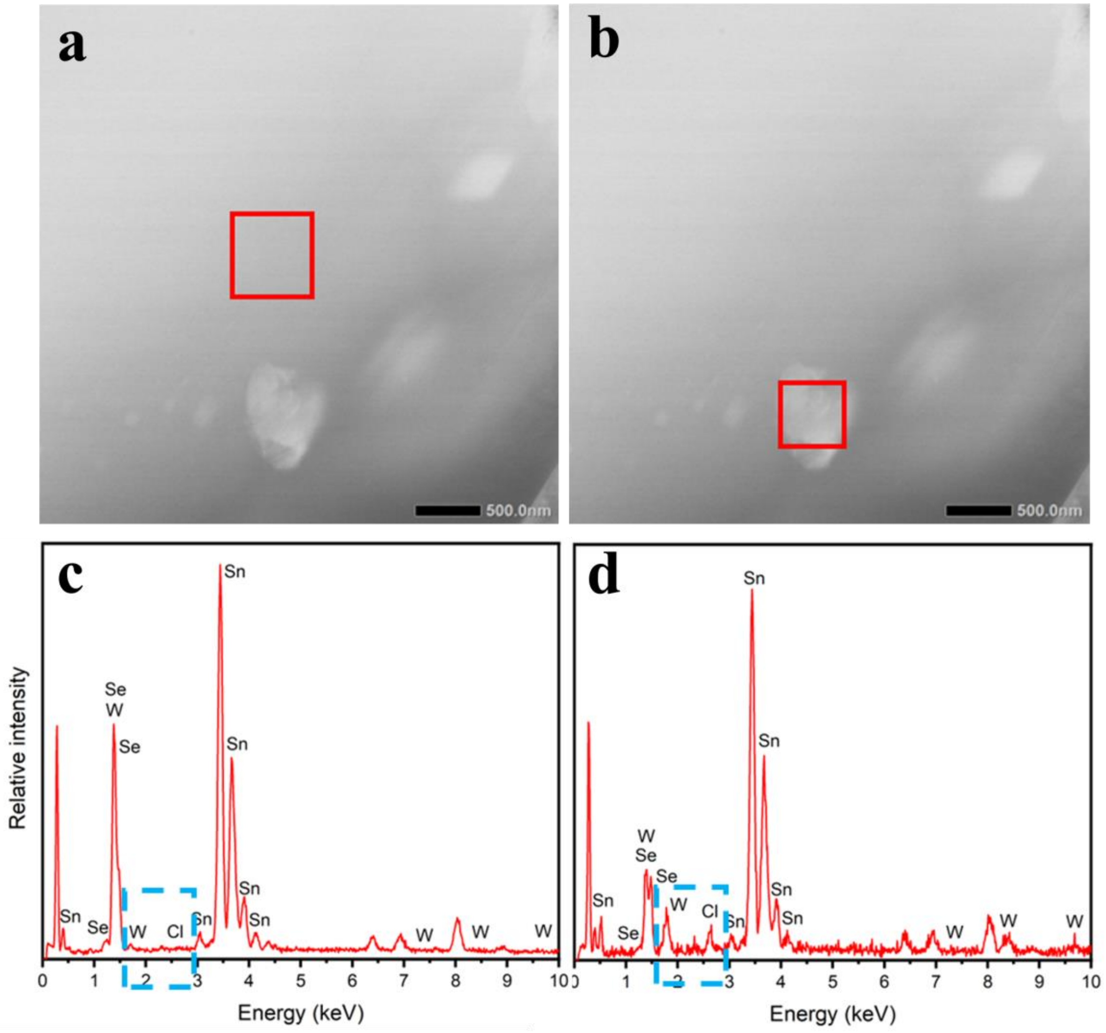

**Supplementary Figure 12.** Repeat measurement results of (**a**) electrical conductivity, (**b**) Seebeck coefficient, (**c**) thermal conductivity, (**d**) *ZT* of $SnSe_{0.92}$ + 0.03 $WCl_6$.

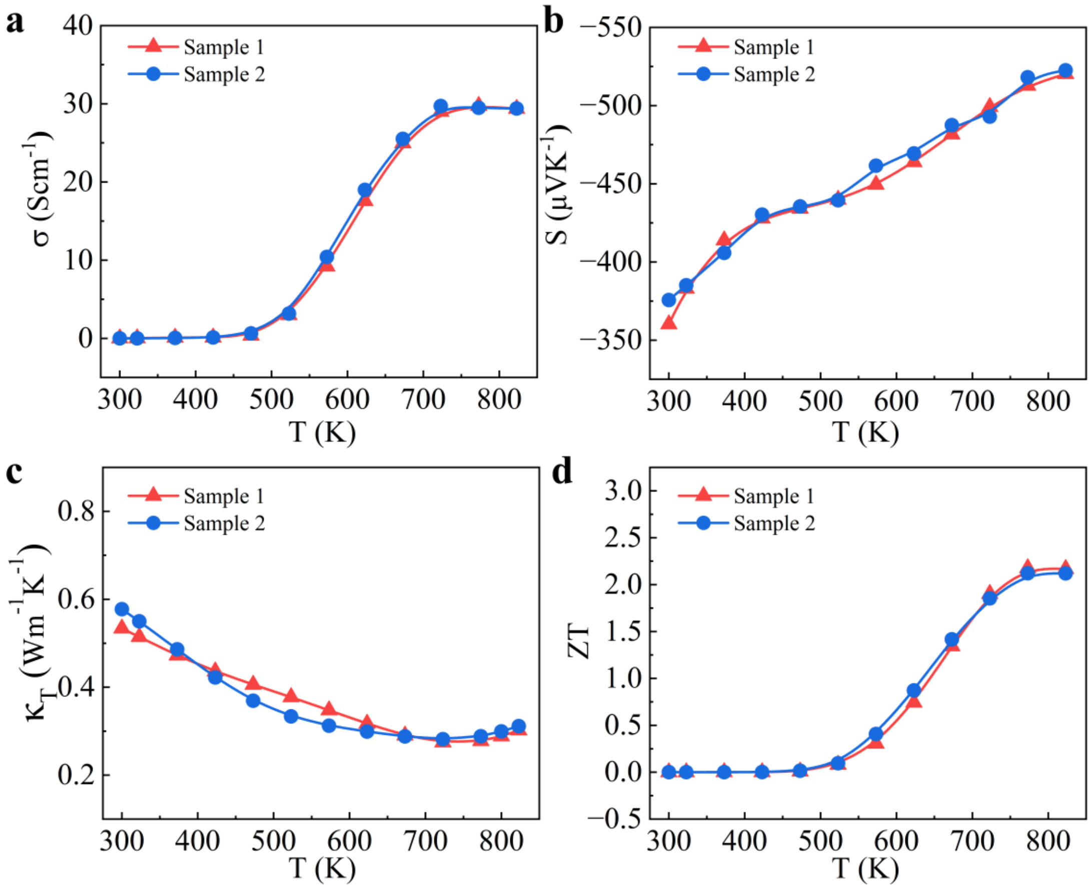

# Reference


1. Zeier W. G. et al. Influence of a Nano Phase Segregation on the Thermoelectric Properties of the p-Type Doped Stannite Compound $Cu_{2+x}Zn_{1-x}GeSe_4$. *J. Am. Chem. Soc.* **134**, 7147-7154 (2012).
2. May A. F. et al. Characterization and Analysis of Thermoelectric Transport in n-Type $Ba_8Ga_{16-x}Ge_{30+x}$. *Phys. Rev. B* **80**, 125205 (2009).
