# Supplementary material for: Divacancy and resonance level enables high thermoelectric performance in n-type SnSe polycrystals
Source: Nat Commun. 2024 May 18;15:4231. doi: 10.1038/s41467-024-48635-0 (PMC11102544; doi:10.1038/s41467-024-48635-0)
Supplement: Supplementary file 3 — Lasing Reporting Summary [file 41467_2024_48635_MOESM3_ESM.pdf]

nature research

Corresponding author(s): Guodong Tang
Haijun Wu
Yongsheng Zhang

Lasing Reporting Summary

Nature Research wishes to improve the reproducibility of the work that we publish. This form is intended for publication with all accepted papers reporting claims of lasing and provides structure for consistency and transparency in reporting. Some list items might not apply to an individual manuscript, but all fields must be completed for clarity.

For further information on Nature Research policies, including our data availability policy, see Authors & Referees.

• Experimental design

Please check: are the following details reported in the manuscript?

1. Threshold

Plots of device output power versus pump power over a wide range of values indicating a clear threshold

☐ Yes
☒ No

This correlation was not addressed in our study.

2. Linewidth narrowing

Plots of spectral power density for the emission at pump powers below, around, and above the lasing threshold, indicating a clear linewidth narrowing at threshold

☐ Yes
☒ No

This correlation was not addressed in our study.

Resolution of the spectrometer used to make spectral measurements

☒ Yes
☐ No

In the Methods section, we describe the experimental parameters used for XPS and Raman testing.

3. Coherent emission

Measurements of the coherence and/or polarization of the emission

☐ Yes
☒ No

This correlation was not addressed in our study.

4. Beam spatial profile

Image and/or measurement of the spatial shape and profile of the emission, showing a well-defined beam above threshold

☐ Yes
☒ No

This correlation was not addressed in our study.

5. Operating conditions

Description of the laser and pumping conditions
Continuous-wave, pulsed, temperature of operation

☐ Yes
☒ No

This correlation was not addressed in our study.

Threshold values provided as density values (e.g. W cm^{-2} or J cm^{-2}) taking into account the area of the device

☐ Yes
☒ No

This correlation was not addressed in our study.

6. Alternative explanations

Reasoning as to why alternative explanations have been ruled out as responsible for the emission characteristics
e.g. amplified spontaneous, directional scattering; modification of fluorescence spectrum by the cavity

☐ Yes
☒ No

This correlation was not addressed in our study.

7. Theoretical analysis

Theoretical analysis that ensures that the experimental values measured are realistic and reasonable
e.g. laser threshold, linewidth, cavity gain-loss, efficiency

☐ Yes
☒ No

This correlation was not addressed in our study.

8. Statistics

Number of devices fabricated and tested

☐ Yes
☒ No

This correlation was not addressed in our study.

Statistical analysis of the device performance and lifetime (time to failure)

☐ Yes
☒ No

This correlation was not addressed in our study.
